# Renal Artery Thrombectomy Causing Functional and Symptomatic Recovery after 50-Hour Delay in Reperfusion of Acute Main Renal Artery Thrombosis

**DOI:** 10.1155/2022/1021683

**Published:** 2022-02-08

**Authors:** Kevin Singh Kang, John Steven Wilson

**Affiliations:** ^1^Department of Cardiology, University of Pittsburgh Medical Center Hamot, Erie, PA 16550, USA; ^2^Department of Cardiology, Meadville Medical Center, Meadville, PA, USA

## Abstract

Acute renal artery thrombosis is rare and even rarer in the thrombus occluding the main renal artery and compromising the entire kidney. We report on a 46-year-old female smoker with no past medical history and no hypercoagulability who developed sudden severe left flank pain, hematuria, acute renal failure, and severe hypertension. A CT angiogram showed totally occluded renal artery at the ostium with a thrombus and severely hypoperfused left kidney with multiple infarcts. Initial course of treatment was with intravenous heparin but with no improvement after 50 hours since symptom onset; angiography was done. This revealed totally occluded renal artery at ostium with no vessels or kidney blush seen. After aspiration thrombectomy, blush was seen in kidney parenchyma along with flow in the arcuate renal arteries although with some distal embolic events. The ostial lesion was treated with a drug eluting stent with excellent result angiographically. However, 8 months later, severe restenosis occurred. This time, the patient did not flank pain or renal failure but had progressive hypertension. The patient was treated this time with rheolytic thrombectomy followed by intravascular ultrasound-guided drug-eluting stenting. The patient has been followed for a year and a half since and recent CT scan revealed widely patent renal arteries bilaterally with normal kidney function, BP, and good perfusion to the left kidney with only tiny areas of infarct. Ultrasound of the kidneys also showed the size of the left kidney as within normal range now, and she has good distal flow velocities in the branch renal arteries. Our case report shows that even delayed reperfusion of complete renal artery occlusion with jeopardized arterial flow to the entire kidney could result in restoration of function to most of the kidney.

## 1. Introduction

Acute renal artery occlusion is rare when involving the entire kidney due to total occlusion of the main renal artery and rarer when due to in situ thrombosis and not embolic event [1]. However, this was noted in our patient as reported here. As expected, acute renal artery occlusion would result in diffuse ischemia and risk of total kidney loss [1, 2]. As noted, embolic events from atrial fibrillation, cardiomyopathy, or valvular heart disease are far more common than in situ thrombosis [1, 3]. In our patient, there were no cardiovascular risk factors of embolic events as confirmed after a two-year follow-up, and clinically and angiographically, in situ thrombus was the most likely scenario. Additionally, the angiographic criteria based on coronary literature were suggestive of plaque rupture and thrombosis [4]. The irregular and ulcerated angiographic appearance in ostial and proximal renal artery suggested in situ thrombosis related to underlying atherosclerotic plaque [4]. The smoking history was the contributor to the plaque rupture appearance and in situ renal artery occlusion.

The diagnosis of acute main renal occlusion is difficult clinically and must be considered in patients presenting with acute renal failure, flank pain, hematuria, high LDH, and new or worsening hypertension even though fever and nausea may be seen also [5, 6]. Often, the complication may arise after renal or aortic endovascular interventions due to dissection [1, 2, 6]. It is clear that if there is acute occlusion of main renal artery, early reperfusion would preserve kidney function but the benefits of late revascularization are unclear [1, 7]. There is also limited information on long-term outcomes for BP and renal function recovery after reperfusion of acute main renal artery thrombosis [1, 7].

Treatment of renal artery thrombosis is initially with anticoagulation, and the benefits are questionable [7]. The more invasive treatments after failure of anticoagulation to improve the patients signs, symptoms, and renal function are anecdotal from small retrospective series or case reports [7]. Often catheter directed thrombolysis is endorsed for the treatment of thrombotic occlusion of the main renal artery but this has been tested mostly in embolic forms of occlusion and not associated with complete restoration of renal function [1]. Since angiography would be mandated for this treatment, and our patient's angiographic as well as clinical characteristics suggested in situ thrombosis, we elected aspiration and rheolytic thrombectomy techniques. There are no data to suggest superiority of catheter directed thrombolysis over thrombectomy techniques [ 7, 8].

## 2. Case Report

**Figure 1:**
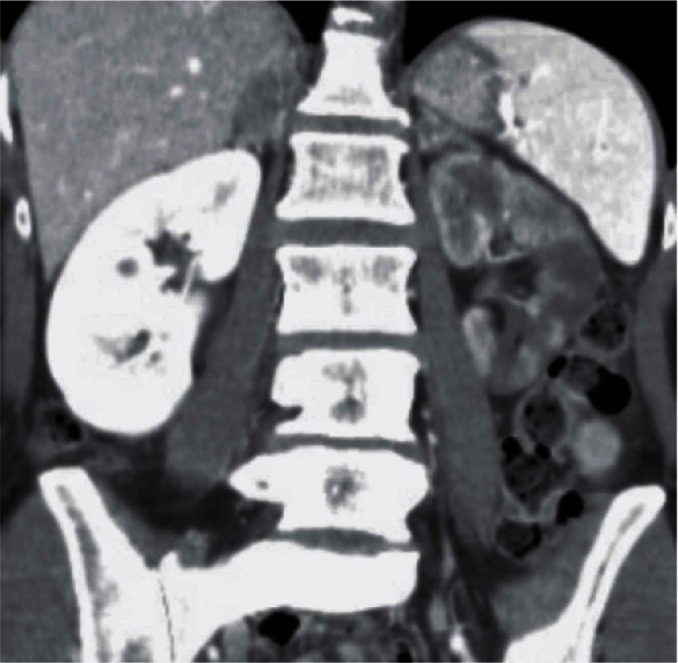
CT angiogram showing normal findings on the right side but the left kidney is severely hypoperfused with probably multiple renal infarcts.

**Figure 2:**
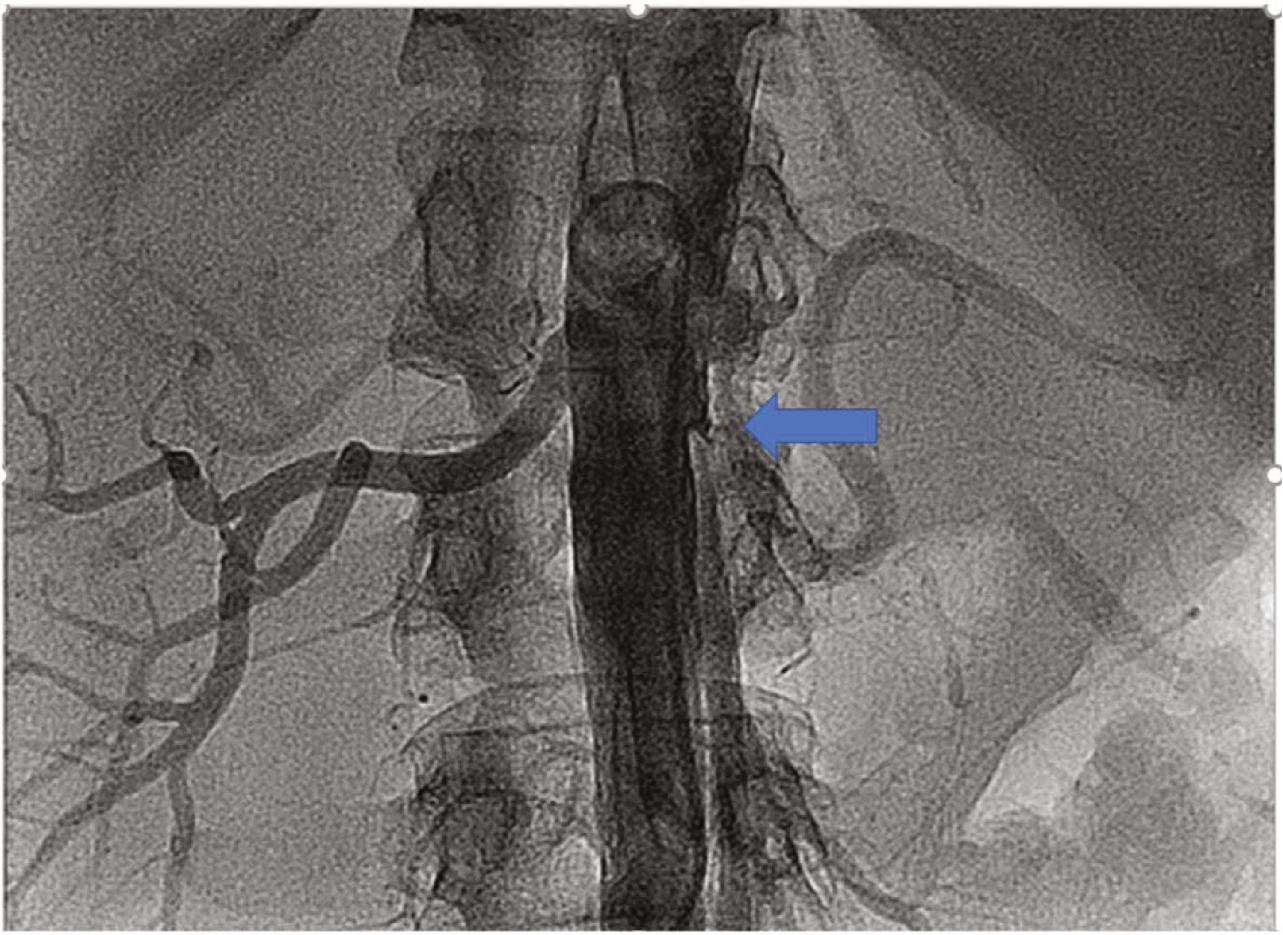
Abdominal aortic angiography revealing normal right-sided findings. In contrast, the left renal artery is totally occluded at the ostium (blue arrow) with angiographic appearance of a thrombus at the ostium with contrast staining.

**Figure 3:**
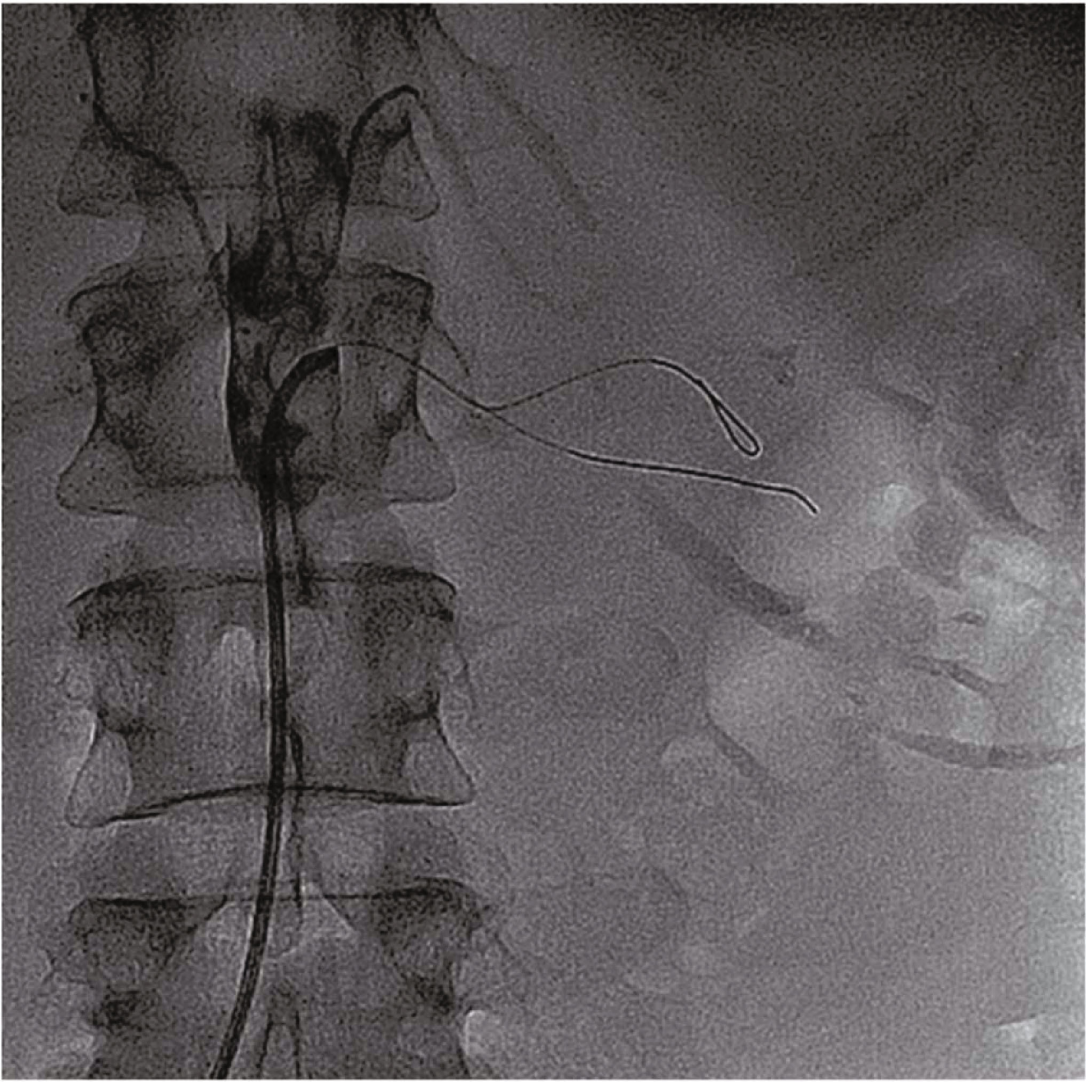
0.014-inch wire across the occlusion still showing no renal blush and no distal arterial vasculature. Second wire placed in the renal artery due to difficulty in advancing the thrombectomy catheter into multiple distal renal artery branches to restore perfusion to multiple renal lobes.

**Figure 4:**
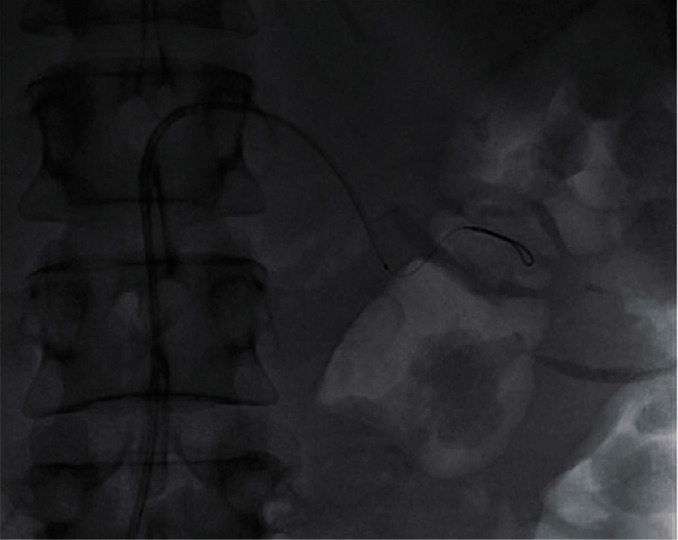
Aspiration thrombectomy catheter taken down multiple interlobar arteries.

**Figure 5:**
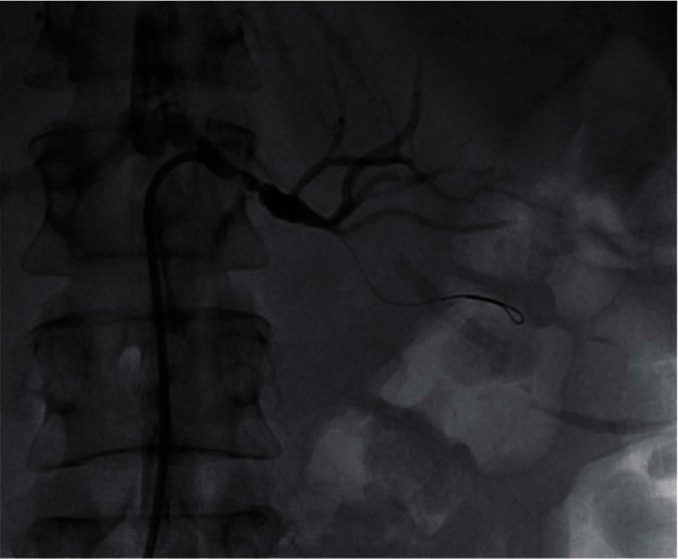
After aspiration thrombectomy, underlying irregular angiographic appearance looks like a plaque rupture at the ostial left renal artery suggesting in situ thrombosis rather than embolism.

**Figure 6:**
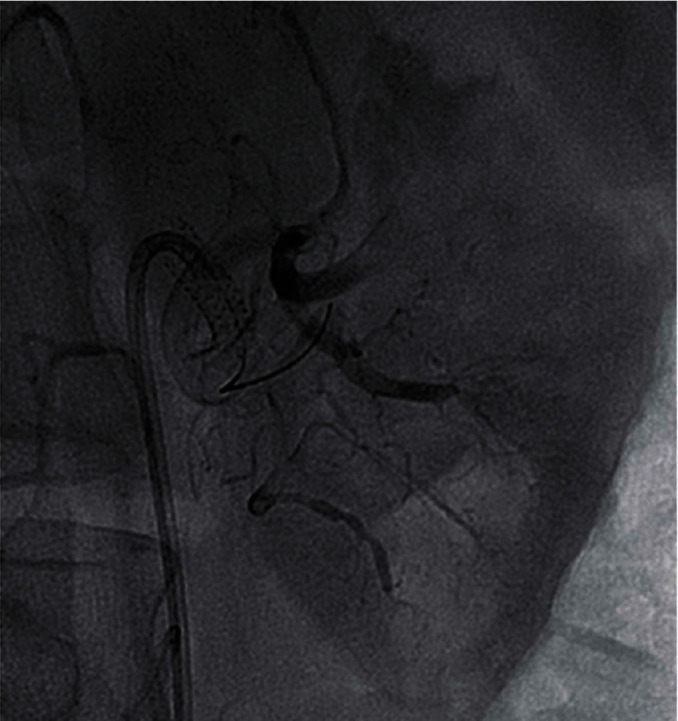
After stenting of the ostium, renal blush is noted.

**Figure 7:**
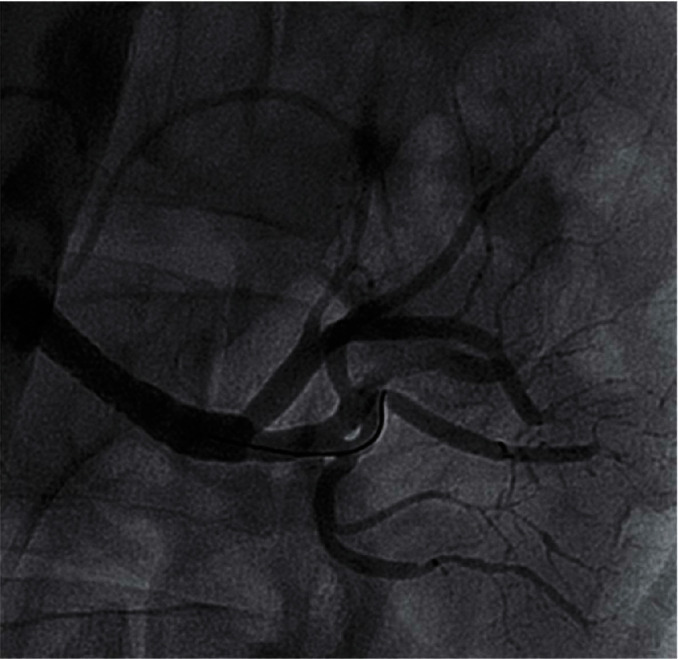
The poststent angiography reveals much better renal perfusion with patent renal lobular and arcuate arteries, but distal vessels still look in spasm and with some distal embolization.

**Figure 8:**
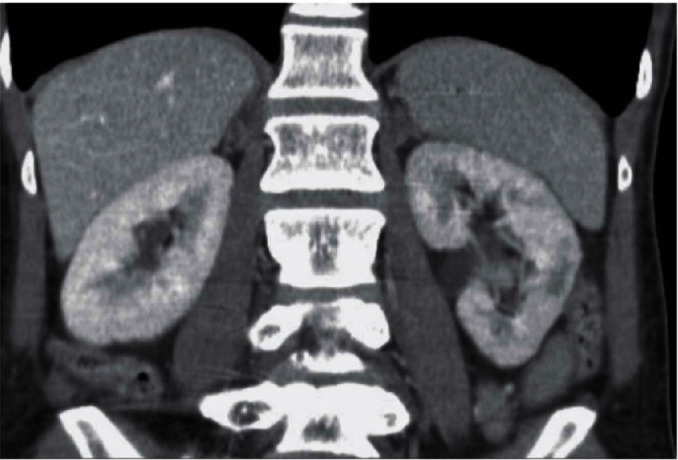
CT angiogram a month later now shows much better renal perfusion with minimal infarcts on the left side, compared to Figure 1.

We report on a 46-year-old female smoker with no past medical history and no hypercoagulability who developed sudden severe left flank pain, hematuria, acute renal failure, and severe hypertension. The symptoms had been going on for about a day prior to presentation. The patient was hypertensive, and the laboratory values showed hematuria and reduced renal function. A CT angiogram showed totally occluded renal artery at the ostium with a thrombus and severely hypoperfused left kidney suggestive of large infarcts in the left kidney (Figure 1). Initial course of treatment was anticoagulation with intravenous heparin but with no improvement over next 24 hours totaling about 48 hours prior to her onset of symptoms without kidney reperfusion. At that point, she was transferred to our hospital. Angiography was done with 6-French sheath in right common femoral artery and 6-French pigtail catheter in the abdominal aorta at the level of renal arteries; this revealed totally occluded renal artery at ostium with no visualization of distal renal arterial tree (Figure 2). There was also no renal blush noted suggestive of renal perfusion from renal artery contrast injections (Figure 3). Selective engagement of left renal artery was done with 6-French internal mammary guiding catheter, and the main renal artery total occlusion at the ostium was crossed with two 0.014-inch nonhydrophilic tipped wires and left in two different lobular branches to allow aspiration thrombectomy of multiple branches (Figure 4). The renal artery thrombus was then treated by aspiration thrombectomy with 6-French-sized Medtronic Xport aspiration catheter with removal of visible clots outside the body (Figure 4). This led to restoration of flow to the kidney, and the ostial lesion looked irregular and had an angiographic appearance of plaque rupture rather than an embolic site which would have resulted in a smooth angiographic appearance (Figure 5). Finally, blush was seen in kidney parenchyma along with flow in the arcuate renal arteries despite a distal embolic event to lobular artery noted by distal vessel cut off (Figure 6). The ostial lesion was treated with a drug eluting stent with excellent result angiographically (Figure 7). The symptoms of severe left flank pain and nausea that were present up to the initiation of procedure resolved completely by the next morning. Blood pressure (BP) was better and within normal range the next day, and the patient was noted to improve renal function. Glomerular filtration rate that was 59 (units are ml/min/1.73 m^2^) on the morning of the procedure improved to 93, the next morning. A month later, CT angiogram showed much better left kidney perfusion, and the kidney was normal in size (Figure 8).

**Figure 9:**
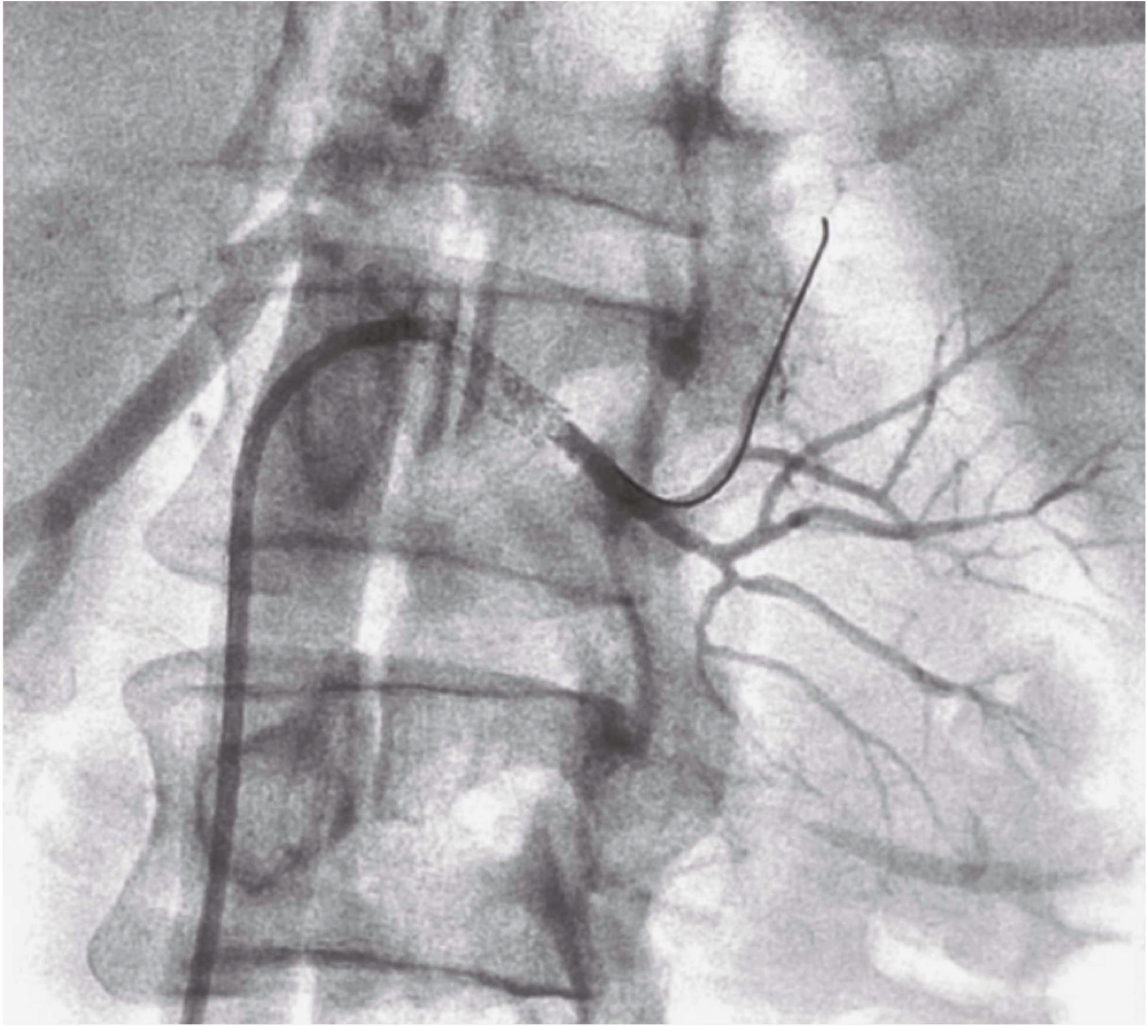
8 months after atherectomy and stenting, angiography shows that the patient has severe instent restenosis with potential thrombus formation as well.

**Figure 10:**
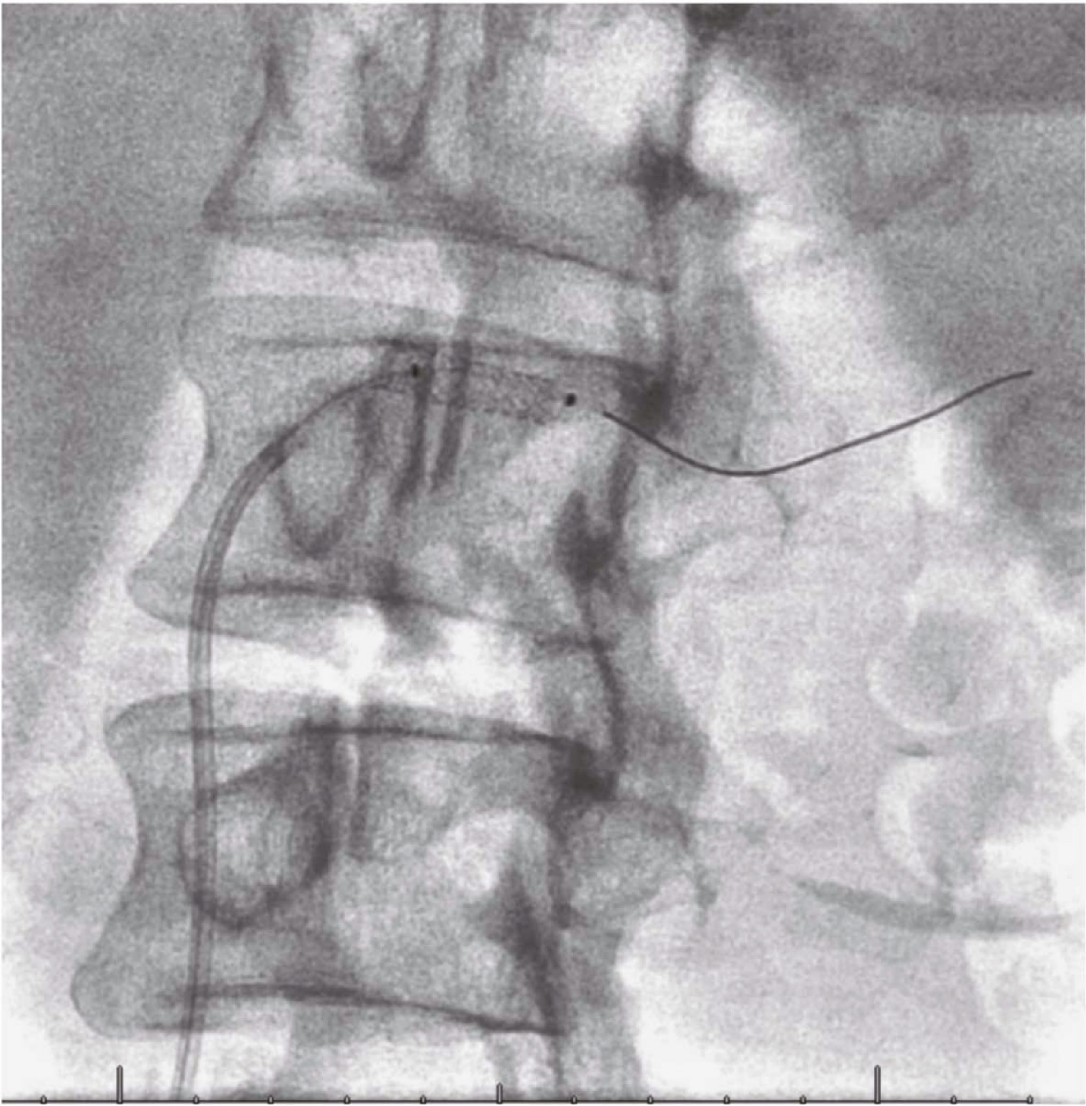
The angiojet thrombectomy was done inside the stent.

**Figure 11:**
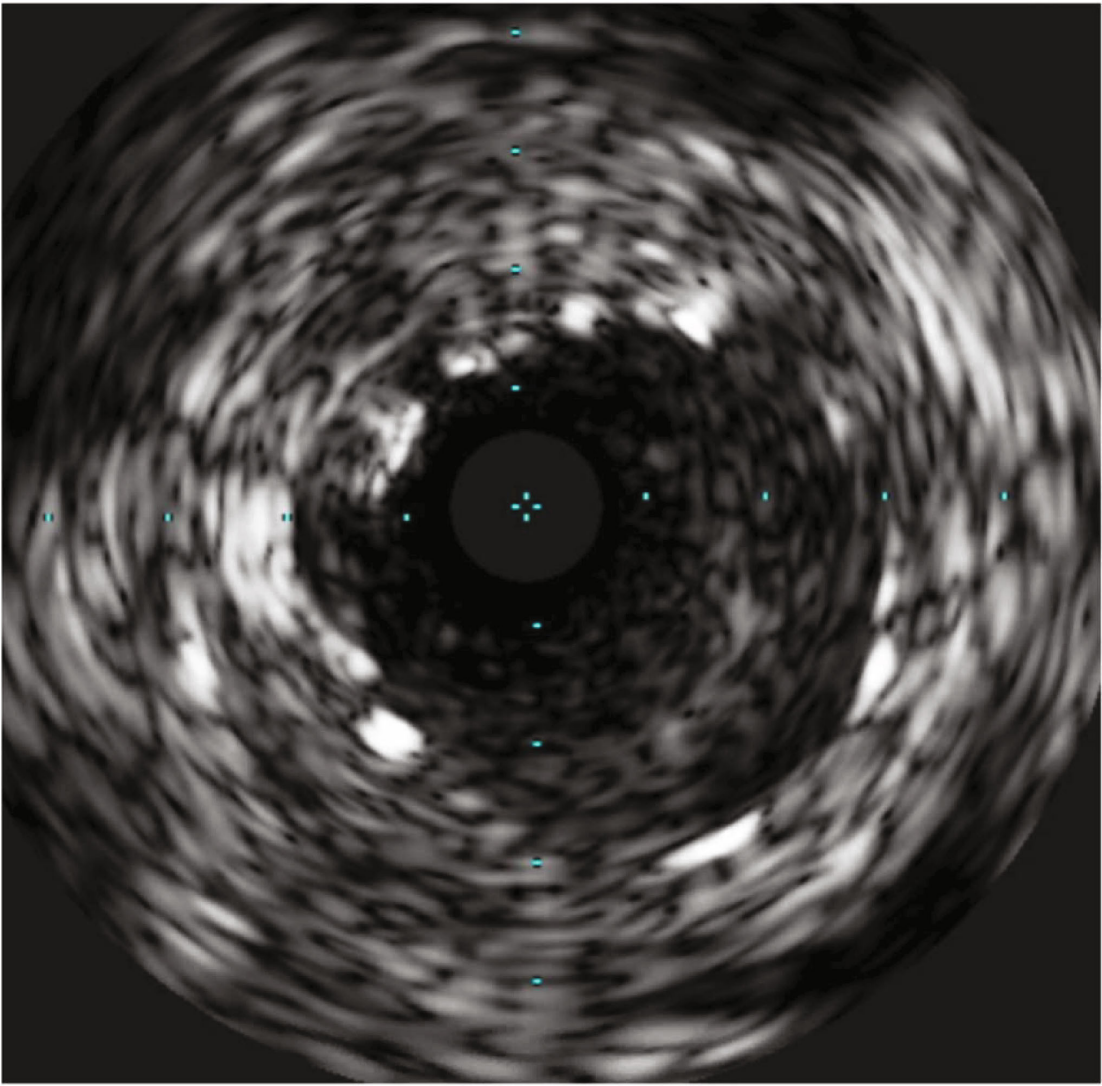
The patient has IVUS view of severe instent restenosis after anjiojet thrombectomy.

**Figure 12:**
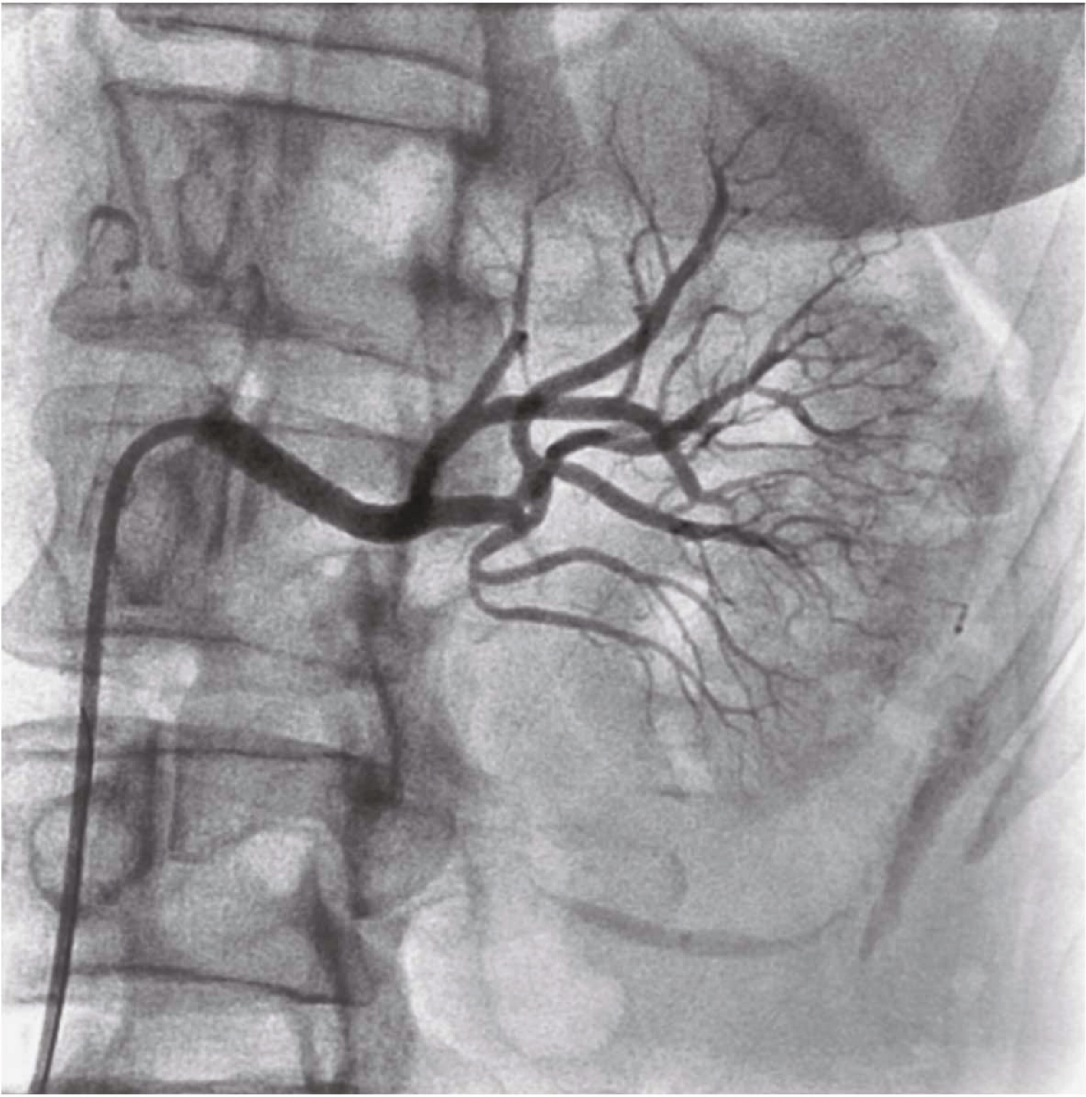
Postrestenting of the renal artery now shows excellent left kidney blush and patent lobular, arcuate and distal kidney vessels with less spasm, and no embolic cut offs, compared to Figure 7.

**Figure 13:**
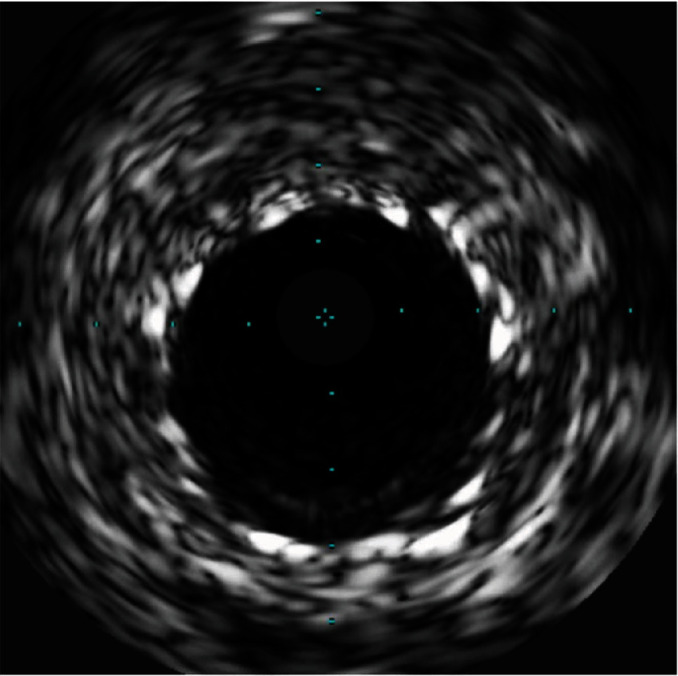
IVUS shows poststent, lumen diameter of over 4 mm with excellent stent expansion.

However, 8 months later, severe restenosis occurred. This time, the patient did not flank pain or renal failure but had progressive hypertension. Ultrasound of the kidney suggested severe left renal instent restenosis, and angiography was done again with the same technique showing severe instent restenosis with mil contrast staining and possible thrombus in the stent (Figure 9). The patient was treated this time with rheolytic thrombectomy using 6-French angiojet device inside the stent (Figure 10). Intravascular ultrasound (IVUS) showed severe instent intimal proliferation (Figure 11). Repeat drug eluting coronary stent was placed with widely patent left renal arterial system angiographically (Figure 12). This was followed by repeat intravascular ultrasound showing widely patent stent lumen (Figure 13).

**Figure 14:**
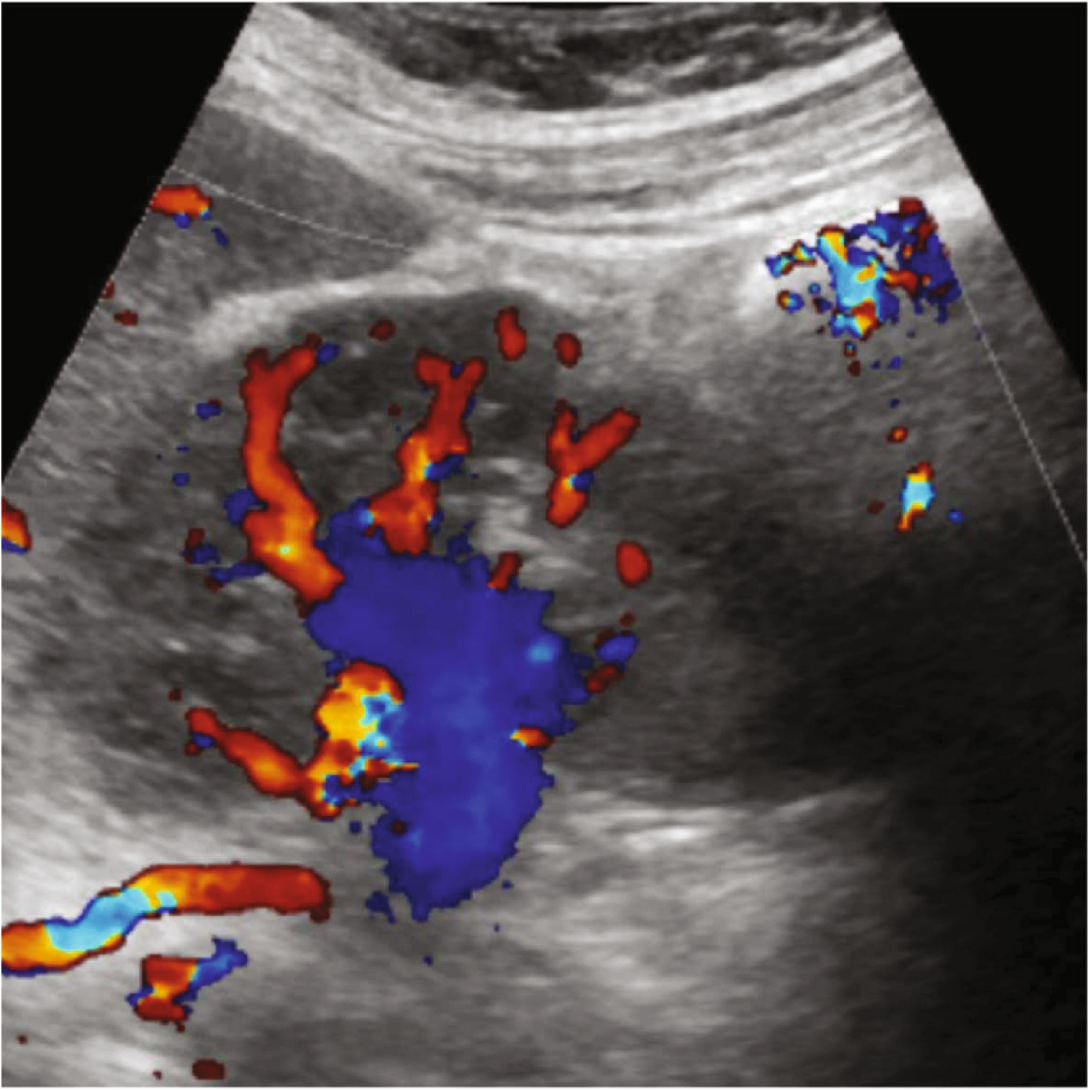
US color Doppler shows patent color flow in distal vessels.

Follow-up CT angiogram had revealed much better perfusion to the left kidney with only a tiny area of infarct. Incidentally a nuclear cardiac stress test that was done with technetium pyrophosphate showed normal perfusion to both kidneys as well. An ultrasound of the kidneys 1 year postinitial presentation had also showed the size of left kidney as within normal range and good distal flow velocities in the branch renal arteries (Figure 14).

The patient has been followed for more than 2 years since her initial event and the recent CT scan revealed widely patent renal arteries bilaterally. On a recent outpatient visit, 49 months after initial presentation, she was asymptomatic with normal creatinine and glomerular filtration rate and with a normal BP on the same antihypertensives that had failed to control her BP in the past.

## 3. Discussion

Acute renal artery thrombosis causing acute renal infarction is a rare clinical syndrome and more often is related to embolic than in situ thrombus [1, 2]. Patients often have risk factors like diabetes, hypertension, spontaneous dissection, fibromuscular dysplasia, or hypercoagulability or may be post an endovascular procedure. Atrial fibrillation is a frequent association causing embolic renal arterial occlusion. Our patient had no prior medical history except for nicotine abuse, and clinical and angiographic findings favored a plaque rupture and in situ thrombosis and hence was treated with thrombectomy and drug eluting stenting [3, 4]. The diagnosis of acute renal artery thrombosis or acute renal infarct is rare and often missed due to rarity of the syndrome [3]. The patient presents often like an acute abdomen with severe abdominal pain and may be confused to have a renal stone like our patient was initially suspected to have nephrolithiasis [5, 6]. There may be acute renal failure and hypertension as in our patient especially if the entire kidney is at risk of infarct and is hypoperfused [1, 7]. There is hematuria, and LDH is high due to renal infarct [1, 5, 6]. Our patient incidentally had all these findings.

The urgency of intervention is not clear at this stage, but the previous case reports have suggested that up to 30 hours of delay in reperfusion of an occluded renal artery may result in salvage of renal function [6, 7].

Diagnosis is often delayed due to rarity of the presentation, but diagnosis can be confirmed by CT angiography (CTA), gadolinium-enhanced MRA, or renal angiography [7]. In our patient, she had a CTA that showed renal artery thrombosis of the main left renal artery at its ostium and severe hypoperfusion of the entire left kidney with initial CTA multiple infarcts in the left kidney.

Goals of therapy are symptom relief, improvement in renal function, and BP control. Most of the available treatments are based on small series or case reports [1, 7]. The initial treatment of acute renal artery thrombosis is anticoagulation but in our patient, anticoagulation with intravenous heparin did not lead to any clinical benefit and resulted in continued symptoms [7]. Systemic thrombolytic therapy has low success and high complication risk in renal arterial thrombosis, and surgical and endovascular options are preferred [7–9]. Surgical treatment includes thrombectomy and aortorenal bypass, but given the high morbidity and mortality of open surgical options, endovascular therapy is preferred [7].

Percutaneous options for in situ or embolic renal artery thromboembolism include intra-arterial thrombolytic and various thrombectomy techniques along with angioplasty and stenting [1, 2, 7]. Thrombectomy techniques, when available, may be preferred given the higher bleeding complications with thrombolytic therapy [7]. Renal artery angioplasty and stenting for thrombosis has been done in case reports [7].

In conclusion, our case report is unusual for using both rheolytic and aspiration thrombectomy as well as stenting and then repeat stenting. It is also unusual for very late reperfusion leading to improvement in symptoms, renal function, and then, improved kidney perfusion on CTA.

## Data Availability

Data is all available at the University of Pittsburgh Medical Center, Hamot.
